# Targeted genomic capture and massively parallel sequencing to identify genes for hereditary hearing loss in middle eastern families

**DOI:** 10.1186/gb-2011-12-9-r89

**Published:** 2011-09-14

**Authors:** Zippora Brownstein, Lilach M Friedman, Hashem Shahin, Varda Oron-Karni, Nitzan Kol, Amal Abu Rayyan, Thomas Parzefall, Dorit Lev, Stavit Shalev, Moshe Frydman, Bella Davidov, Mordechai Shohat, Michele Rahile, Sari Lieberman, Ephrat Levy-Lahad, Ming K Lee, Noam Shomron, Mary-Claire King, Tom Walsh, Moien Kanaan, Karen B Avraham

**Affiliations:** 1 Department of Human Molecular Genetics and Biochemistry, Sackler Faculty of Medicine, Tel Aviv University, Tel Aviv 69978, Israel; 2 Department of Biological Sciences, Bethlehem University, Bethlehem, Palestinian Authority; 3 Genome High-Throughput Sequencing Laboratory, Tel Aviv University, Tel Aviv 69978, Israel; 4 Institute of Medical Genetics, Wolfson Medical Center, Holon 58100, Israel; 5 Genetics Institute, Ha'Emek Medical Center, Afula 18341, Israel; 6 Rappaport Faculty of Medicine, Technion-Israel Institute of Technology, Haifa 32000, Israel; 7 Danek Gartner Institute of Human Genetics, Sheba Medical Center, Tel Hashomer 52621, Israel; 8 Department of Medical Genetics, Rabin Medical Center, Beilinson Campus, Petah Tikva, Israel; 9 Darr Al Kalima Audiological Clinic, Bethlehem, Palestinian Authority; 10 Medical Genetics Institute, Shaare Zedek Medical Center, Jerusalem 91031, Israel; 11 Hebrew University Medical School, Jerusalem 91120, Israel; 12 Department of Medicine (Medical Genetics) and Department of Genome Sciences, University of Washington, Seattle, WA 98195, USA; 13 Department of Cell and Developmental Biology, Sackler Faculty of Medicine, Tel Aviv University, Tel Aviv 69978, Israel

## Abstract

**Background:**

Identification of genes responsible for medically important traits is a major challenge in human genetics. Due to the genetic heterogeneity of hearing loss, targeted DNA capture and massively parallel sequencing are ideal tools to address this challenge. Our subjects for genome analysis are Israeli Jewish and Palestinian Arab families with hearing loss that varies in mode of inheritance and severity.

**Results:**

A custom 1.46 MB design of cRNA oligonucleotides was constructed containing 246 genes responsible for either human or mouse deafness. Paired-end libraries were prepared from 11 probands and bar-coded multiplexed samples were sequenced to high depth of coverage. Rare single base pair and indel variants were identified by filtering sequence reads against polymorphisms in dbSNP132 and the 1000 Genomes Project. We identified deleterious mutations in *CDH23, MYO15A, TECTA, TMC1*, and *WFS1*. Critical mutations of the probands co-segregated with hearing loss. Screening of additional families in a relevant population was performed. TMC1 p.S647P proved to be a founder allele, contributing to 34% of genetic hearing loss in the Moroccan Jewish population.

**Conclusions:**

Critical mutations were identified in 6 of the 11 original probands and their families, leading to the identification of causative alleles in 20 additional probands and their families. The integration of genomic analysis into early clinical diagnosis of hearing loss will enable prediction of related phenotypes and enhance rehabilitation. Characterization of the proteins encoded by these genes will enable an understanding of the biological mechanisms involved in hearing loss.

## Background

Clinical diagnosis is the cornerstone for treatment of human disease. Elucidation of the genetic basis of human disease provides crucial information for diagnostics, and for understanding mechanisms of disease progression and options for treatment. Hence, determination of mutations responsible for genetically heterogeneous diseases has been a major goal in genomic medicine. Deafness is such a condition, with 61 nuclear genes identified thus far for non-syndromic sensorineural hearing impairment [1] and many more for syndromes including hearing loss. Despite the very rapid pace of gene discovery for hearing loss in the past decade, its cause remains unknown for most deaf individuals.

Most early-onset hearing loss is genetic [2]. Of genetic cases, it is estimated that approximately 30% are syndromic hearing loss, with nearly 400 forms of deafness associated with other clinical abnormalities, and approximately 70% are non-syndromic hearing loss, where hearing impairment is an isolated problem [3]. Today, most genetic diagnosis for the deaf is limited to the most common mutations in a patient's population of origin. In the Middle East, these include specific mutations in 9 genes for hearing loss in the Israeli Jewish population [4] and in 13 genes in the Palestinian Arab population [5-7]. As elsewhere, the most common gene involved in hearing loss in the Middle East is *GJB2*, which is responsible for 27% of congenital hearing loss among Israeli Jews [4] and 14% of congenital hearing loss among Palestinian Arabs [5]. Each of the other known genes for hearing loss is responsible for only a small proportion of cases. The large number of these genes, as well as in some cases their large size, has heretofore precluded comprehensive genetic diagnosis in these populations. Using targeted DNA capture and massively parallel sequencing (MPS), we screened 246 genes known to be responsible for human or mouse deafness in 11 probands of Israeli Jewish and Palestinian Arab origin and identified mutations associated with hearing loss in a subset of our probands and their extended families.

## Results

### Targeted capture of exons and flanking sequences of 246 genes

We developed a targeted capture pool for identifying mutations in all known human genes and human orthologues of mouse genes responsible for syndromic or non-syndromic hearing loss. Targets were 82 human protein-coding genes, two human microRNAs and the human orthologues of 162 genes associated with deafness in the mouse (Additional file 1). The Agilent SureSelect Target Enrichment system was chosen to capture the genomic regions harboring these genes, based on the hybridization of complementary custom-designed biotinylated cRNA oligonucleotides to the target DNA library and subsequent purification of the hybrids by streptavidin-bound magnetic bead separation [8]. The UCSC Genome Browser hg19 coordinates of the 246 genes were submitted to the eArray website to design 120-mer biotinylated cRNA oligonucleotides that cover all exons, both coding and untranslated regions (UTRs), and for each exon, 40 flanking intronic nucleotides (Additional file 2). A 3 × centered tiling design was chosen and the repeat masked function was used to avoid simple repeats [9]. A maximum 20-bp overlap into repeats was allowed in order to capture small exons that are closely flanked on one or both sides by short interspersed elements (SINEs). Segmentally duplicated regions were not excluded because this would preclude identifying causative alleles in genes such as *STRC *[10] and *OTOA *[5]. The entire design, across 246 loci, spanned 1.59 Mb. Approximately 8% of targeted regions failed probe design because of proximity of simple repeats. The final capture size was 1.43 MB, including 31,702 baits used to capture 3,959 regions comprising 3,981 exons. Paired-end libraries were created from genomic DNA samples from peripheral blood of 11 probands of families with hearing loss (Table 1) and hybridized with the cRNA capture oligonucleotides.

**Table 1:** Numbers of rare variants detected in genomic DNA of probands with hearing loss

		Rare variants			Potentially functional variants				
		
Proband	Inheritance	SNP	Private indels	Total	Nonsense	Missense^a^	Splice junctions	Frameshift	Total
D28C	Recessive	24	14	38	0	9	0	0	9
Z686A	Recessive	17	13	30	0	7	1	0	8
Z421A	Recessive	20	8	28	0	6	1	1	8
K13576A	Dominant	31	42	73	0	13	1	0	14
W1098A	Dominant	38	8	46	0	15	2	0	17
DC5	Recessive	21	47	68	0	5	0	0	5
DQ3	Recessive	26	58	84	0	11	0	0	11
DR3	Recessive	18	60	78	1	6	1	0	8
CJ3	Recessive	19	52	71	0	3	0	0	3
CK3	Recessive	29	61	90	0	9	1	0	10
DE3	Recessive	26	53	79	1	8	1	0	10

^a^Missense variants predicted to be benign by PolyPhen2 and SIFT are excluded from the missense mutations listed above.

### Massively parallel sequencing of DNA libraries from probands

The captured DNA library from each proband was labeled with a different 6-mer barcode, and the multiplexed libraries (one to two libraries per lane) were analyzed with paired-end sequencing at a read length of 2 × 72 bp, using the Illumina Genome Analyzer IIx. Across the 1.43 MB of captured targets, median base coverage was 757 × to 2,080 ×, with 95% and 92% of targeted bases covered by more than 10 or 30 reads, respectively. We aligned reads to the human reference genome sequence (hg19) and generated SNP and indel calls for all samples. Rare variants were identified by filtering against dbSNP132, the 1000 Genomes project and additional filters (described in the Bioinformatics section of Materials and methods) and classified by predicted effect on the protein, as described in Materials and methods.

### Discovery of novel mutations

In each of the 11 probands, multiple potentially functional variants of predicted damaging effect were identified by our approach and validated by Sanger sequencing (Table 1). Each validated variant was tested for co-segregation with hearing loss in the proband's family. Only the variants reported below were co-inherited with hearing loss.

#### TMC1

**Figure 1:**
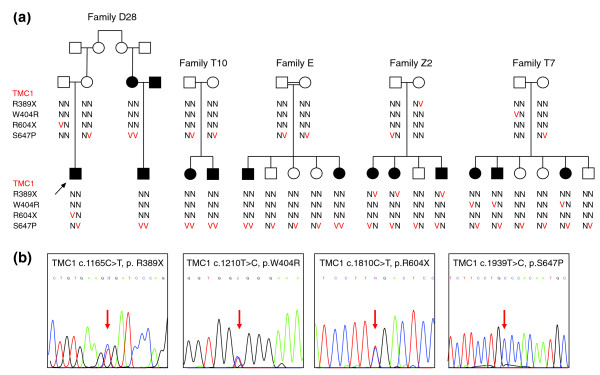
**Pedigrees of families with *TMC1 *mutations**. **(a) **TMC1 p.R604X and p.S647P were discovered by targeted capture and MPS. TMC1 p.R389X and p.W404R were subsequently identified in probands heterozygous for one of the first two alleles. Segregation of alleles with hearing loss is indicated by wild-type (N) and deafness-associated variants (V). The black arrow indicates the proband in each family. **(b) **Sanger sequences of each variant for representative homozygous or heterozygous individuals. The red arrow indicates the mutation.

Family D28 is of Jewish Moroccan ancestry, now living in Israel. Four family members with profound hearing loss consistent with autosomal recessive inheritance were enrolled in the study (Figure 1). In genomic DNA from the proband D28C, two variants were observed in the *TMC1 *gene, corresponding to the known mutation c.1810C > T, p.R604X [11] and a novel variant c.1939T > C, p.S647P (Table 2). Variant reads were 51% and 48% of total reads, suggesting heterozygosity for both alleles. *TMC1*, specifically expressed in the cochlea, encodes a transmembrane channel protein, and is a known gene for hearing loss [12,13]. TMC1 p.S647P is located in the sixth TMC1 transmembrane domain at a fully conserved site and is predicted to be damaging by PolyPhen2 and SIFT.

**Table 2:** Mutations identified by targeted capture and MPS in families with non-syndromic hearing loss

Proband	Inheritance	Genomic coordinates^a^	Reference reads	Variant reads	Total reads	Gene	cDNA (RefSeq ID)	Protein (RefSeq ID)	PolyPhen-2 HumVar score
D28C	Recessive	chr9:75435804 C > T	643	666	1,309	*TMC1 *	c.1810C > T (NM_138691)	p.R604X (NP_619636)	Nonsense
		chr9:75435933 T > C	770	707	1,477	*TMC1 *	c.1939T > C (NM_138691)	p.S647P (NP_619636)	0.912
Z686A	Recessive	chr10:73565593 G > T	0	425	425	*CDH23 *	c.7903G > T (NM_022124.5)	p.V2635F (NP_071407)	0.876
Z421A	Recessive	chr17:18058028 G > A	43	43	86	*MYO15A *	c.8183G > A (NM_016239)	p.R2728H (NP_057323)	0.992
		chr17:18022487 delCG^b^				*MYO15A *	c.373delCG (NM_016239)	p.R125VfsX101 (NP_057323)	Frameshift
DC5	Recessive	chr17:1,035800 G > A	0	89	89	*MYO15A *	c.4240G > A (NM_016239)	p.E1414K (NP_057323)	0.971
K13576A	Dominant	chr4:6304112 G > A	86	90	176	*WFS1 *	c.2756G > A (NM_001145853)	p.E864K (NP_001139325)	0.959
W1098A	Dominant	chr11:121038773 C > T	855	808	1,663	*TECTA *	c.5597C > T (NM_005422.2)	p.T1866M (NP_005413)	0.995

^a^hg19. ^b^Detected as two SNPs by MPS (see explanation in text).

TMC1 p.S647P appears to be a founder mutation for hearing loss in the Moroccan Jewish population. The Moroccan Jewish community is an ancient population that until recently was highly endogamous. In our cohort, among 52 Moroccan Jewish individuals with hearing loss, not closely related to each other by self-report, 10 were homozygous for CX26 c.35delG, 10 were homozygous for TMC1 p.S647P, 6 were compound heterozygous for TMC1 p.S647P and p.R604X, and 9 were heterozygous for TMC1 p.S647P. The allele frequency of TMC1 p.S647P in this series of Moroccan Jewish deaf is therefore (20 + 6 + 9)/104, or 0.34 (Table 3). In contrast, among 282 hearing controls of Moroccan Jewish ancestry, 16 were heterozygous for p.S647P and none were homozygous, yielding an allele frequency estimate of 16/564, or 0.028, and a carrier frequency of 5.7%. The difference between p.S647P allele frequencies in cases and controls was significant at *P *< 10^-23^. TMC1 p.S647P was not detected among 121 deaf probands or 138 hearing controls of other Israeli Jewish ancestries.

**Table 3:** Allele frequency among unrelated deaf and controls of the same population of origin as the proband

			Allele frequency in population of origin (number of chromosomes)	
			
Gene	Mutation	Origin of proband	Unrelated deaf (sample size)	Controls (sample size)
*TMC1*	p.R604X	Morocco, Jewish	0.058 (6/104)	0 (0/256)
*TMC1*	p.S647P	Morocco, Jewish	0.337 (35/104)	0.028 (16/564)
*CDH23*	p.V2635F	Algeria, Jewish	0.192 (5/26)	0 (0/106)
*MYO15A*	p.R2728H	Ashkenazi Jewish	0.010 (3/288)	0 (0/316)
*MYO15A*	p.R125VfsX101	Ashkenazi Jewish	0.008 (1/120)	0.006 (3/480)
*MYO15A*	p.E1414K	Palestinian Arab	0.005 (2/434)	0 (0/480)
*WFS1*	p.E864K	Ashkenazi Jewish	0 (0/102)	0 (0/100)
*TECTA*	p.T1866M	Turkey, Jewish	0.067 (1/15)	0 (0/270)

Sanger sequencing of the entire coding region of *TMC1 *in genomic DNA of the seven probands heterozygous for TMC1 p.S647P revealed *TMC1 *c.1165C > T, p.R389X [14] as the second pathogenic allele in two probands. In two other probands heterozygous for TMC1 p.S647P, the novel variant *TMC1 *c.1210T > C, p.W404R, with PolyPhen2 score 0.567, was revealed as a possible second pathogenic allele (Figure 1). Neither TMC1 p.R389X nor TMC1 p.W404R were found in an additional 51 Moroccan deaf probands or 82 Moroccan Jewish controls. We estimate that *TMC1 *mutations explain at least 38% of inherited hearing loss in the Moroccan Jewish population.

#### CDH23

**Figure 2:**
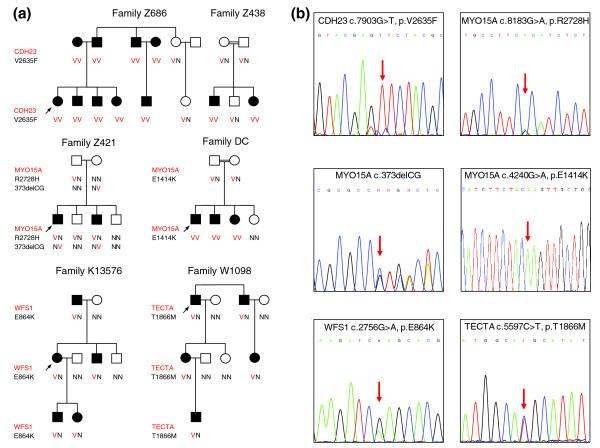
**Pedigrees of families with *CDH23, MYO15A, TECTA*, and *WFS1 *mutations**. **(a) **Segregation of hearing loss with wild-type (N) and deafness-associated variants (V) in each family. **(b) **Sanger sequences of each variant.

Family Z686 is of Jewish-Algerian descent, now living in Israel. Nine family members with profound hearing loss and two relatives with normal hearing enrolled in the study (Figure 2). Hearing loss in the family is consistent with autosomal recessive inheritance. In genomic DNA from proband Z686A, a novel variant in *CDH23 *was observed in 100% of reads, indicating homozygosity (Table 2). This variant corresponds to *CDH23 *c.7903G > T, p.V2635F and co-segregates perfectly with hearing loss in the extended kindred (Figure 2). CDH23 p.V2635F is predicted to be damaging by PolyPhen2 and SIFT. The *CDH23 *mutation was screened in hearing controls and deaf probands of Jewish origin (Table 3). Proband Z438A, of Algerian origin, was homozygous for the mutation, which segregated with hearing loss in his family. Another deaf proband with partial Algerian ancestry, D16C, was heterozygous for CDH23 p.V2635F. All 68 exons of *CDH23 *were sequenced in genomic DNA of D16C, but no second mutation was detected. D16C may be a carrier of CDH23 p.V2635F, with his hearing loss due to another gene.

#### MYO15A

Family Z421 is of Jewish Ashkenazi origin. Hearing loss in the family is consistent with recessive inheritance (Figure 2). The proband is heterozygous for two novel variants in *MYO15A *(Tables 2 and 3). The first variant, corresponding to *MYO15A *c.8183G > A (p.R2728H), was supported by 50% (43/86) of reads and is predicted to be damaging by PolyPhen2 and SIFT. The other *MYO15A *variant was cryptic. It was read as two single base-pair substitutions 2 bp apart, at chr17:18,022,486 C > G and chr17:18,022,488 G > C, but each variant was supported by only 25% of reads. In our experience, two apparently adjacent or nearly adjacent single base-pair variants with similar numbers of reads, each with weak support, may reflect an underlying insertion or deletion. We sequenced *MYO15A *exon 2 containing these variant sites and detected a 2-bp deletion *MYO15A *c.373delCG (p.R125VfsX101). MYO15A p.R2728H and *MYO15A *c.373delCG co-segregated with hearing loss in the family. *MYO15A*, which encodes a myosin expressed in the cochlea, harbors many mutations worldwide responsible for hearing loss [15,16], but neither MYO15A p.R2728H nor *MYO15A *c.373delCG has been described previously.

Family DC is of Palestinian Arab origin. Hearing loss in the family is congenital, profound, and recessive (Figure 2). The proband is homozygous for *MYO15A *c.4240G > A (p.E1414K), a novel mutation predicted to be damaging by Polyphen2 and SIFT (Tables 2 and 3).

#### WFS1

Family K13576 is of Ashkenazi Jewish origin. Hearing loss in the family is dominant (Figure 2). Audiograms of affected relatives reveal hearing thresholds in a U-shaped pattern, with poorest hearing in low and middle frequencies. The proband is heterozygous for missense mutation *WFS1 *c.2765G > A (p.E864K) (Tables 2 and 3). *WFS1 *encodes wolframin. Homozygosity for this mutation is known to cause Wolfram syndrome, which includes optic atrophy and non-insulin-dependent diabetes mellitus (MIM ID 606201.0020) [17,18]. Heterozygosity for this mutation is responsible for non-syndromic low-frequency hearing loss in a Japanese family [19] with a similar phenotype to that of family K13576.

#### TECTA

Family W1098 is of Turkish Jewish descent. Hearing loss in the family is dominant (Figure 2). The critical mutation in the proband is *TECTA *c.5597C > T (p.T1866M) (Tables 2 and 3), which encodes alpha-tectorin [20]. Heterozygosity at this allele has been associated with dominantly inherited hearing loss in other families [21,22].

In addition to the probands described above, in five other probands of Palestinian Arab origin (DR3, DE5, DQ3, CJ3 and CK3), multiple variants were identified by capture and sequencing, and validated by Sanger sequencing, but none co-segregated with hearing loss in the families (Table 1). In these families, hearing loss could be due to mutations in non-captured regions of genes in our pools or by as-yet-unknown genes.

## Discussion

The goal of our study was to apply DNA capture and MPS to identify inherited mutations involved in hearing loss. We designed oligonucleotides to capture the exons and regulatory regions of 246 genes involved in hearing loss, in human or in mouse. The inclusion of genes thus far known to be involved in deafness in the mouse is based on the observation that many genes for human deafness are responsible for mouse deafness as well [23,24]. Among the genes harboring mutations causing deafness only in the mouse, no deleterious mutations were present in these 11 human families. The mouse genes will be sequenced from DNA of many more human families in the future.

Comprehensive targeted enrichment and MPS has been employed previously for non-syndromic hearing loss [25]. Our approach targeted more genes (246 versus 54), including in particular genes associated with deafness in the mouse. Our goal in including these genes is to speed future discovery of additional human deafness genes that are orthologues of known mouse genes.

To date, routine clinical diagnostic tests for deafness in the Middle East have consisted of restriction enzyme analysis of the two common *GJB2 *mutations, and on occasion, DNA sequencing of the *GJB2 *coding region. In some clinics, screening for the relevant mutations in other genes on the basis of ethnic origin, audiological tests, family history, personal history and findings from physical examination may be performed. Comprehensive testing for genes with mutations common in other populations, such as *TMC1 *[11,26], *MYO15A *[15] or *SLC26A4 *[27], is not available from health services in the Middle East due to the high cost of testing these genes by Sanger sequencing. The large size of these genes has also precluded their analysis in Middle Eastern research laboratories.

A major challenge for mutation discovery is determining which variants are potentially causative and which are likely benign. This is particularly difficult when sequencing populations that are not well represented in dbSNP. A novel variant may represent a previously undiscovered common population-specific polymorphism or a truly private mutation. Sequencing even a small number of samples (say 100) from the same ethnic background serves as a very effective filter. In our study, many variants not in dbSNP were nonetheless common in our populations and could be ruled out as causative mutations (Additional file 3). As a result, a smaller fraction of the detected variants had to be verified by Sanger sequencing for segregation in the family.

For the Israeli deaf population of Moroccan Jewish ancestry, this study has substantial clinical implications, as the *TMC1 *gene was found to be very frequently involved in deafness in this population. Recessive mutations in *TMC1 *were detected in more than a third (38%) of hearing impaired Jews of Moroccan origin. A single DNA sample of a Moroccan Jewish proband, evaluated by this approach, led to the discovery of four mutations, two of them novel, and solved the cause of hearing loss of an additional 20 families. The *TMC1 *gene is the sixth most common cause of recessive hearing loss worldwide [27]. The two novel mutations in Moroccan Jewish deaf individuals add to the 30 recessive mutations that have been reported to date in the *TMC1 *gene [27]. In some populations, including Iran [26] and Turkey [11], as Israel, *TMC1 *is one of the genes most frequently involved in deafness. Based on these results, we recommend that all Israeli Jewish probands of Moroccan ancestry be screened for the four *TMC1 *mutations, as well as for the most common *GJB2 *mutations, prior to conducting MPS. An immediate result of these findings is that screening for *TMC1 *mutations will become routine in Israel for all hearing impaired patients of Moroccan Jewish ancestry.

Novel mutations were identified in multiple other genes - *CDH23, MYO15A, WFS1*, and *TECTA *- that are known to be responsible for hearing loss but are not routinely evaluated, largely because of their size. Targeted MPS makes it feasible to screen large genes that have heretofore been largely untested. As sequencing chemistry improves, we believe it will be feasible to multiplex 12 samples per lane and still maintain a high coverage (> 200 ×). It will thus become even more straightforward to screen comprehensively for all known hearing loss genes.

Of the six Palestinian families enrolled in this study, a causative mutation was found in only one. This result is probably due to two factors. First, familial hearing loss in the Palestinian population has been very thoroughly investigated for more than a decade, with the discovery of many critical genes and the characterization of the mutational spectra of these genes as they were identified (for example, [5,7,28,29]). Therefore, the mutations responsible for hearing loss in many Palestinian families were known before this project was undertaken. Second, as the result of historical marriage patterns, inherited hearing loss in the Palestinian population is likely to be more heterogeneous, at the levels of both alleles and loci, than is inherited hearing loss in the Israeli population. A large proportion of Palestinian families are likely to have hearing loss due to as yet unknown genes. Since the molecular basis of deafness in most of our Palestinian probands was unsolved, we predict that many new genes for hearing loss remain to be found. These may be optimally resolved by exome sequencing in combination with homozygosity mapping, as we previously demonstrated [6].

## Conclusions

Multiple mutations responsible for hearing loss were identified by the combination of targeted capture and MPS technology. Screening multiple families for alleles first identified in one proband led to the identification of causative alleles for deafness in a total of 25 of 163 families. The approach described here exploits the high throughput of targeted MPS to make a single fully comprehensive test for all known deafness genes. Although we applied it within the context of familial hearing loss, the test could also be used in cases of isolated deafness. This strategy for clinical and genetic diagnosis will enable prediction of phenotypes and enhance rehabilitation. Characterization of the proteins encoded by these genes will enable a comprehensive understanding of the biological mechanisms involved in the pathophysiology of hearing loss.

## Materials and methods

### Family ascertainment

The study was approved by the Helsinki Committees of Tel Aviv University, the Israel Ministry of Health, the Human Subjects Committees of Bethlehem University, and the Committee for Protection of Human Subjects of the University of Washington (protocol 33486). Eleven probands and both affected and unaffected relatives in their families were ascertained. A medical history was collected, including degree of hearing loss, age at onset, evolution of hearing impairment, symmetry of the hearing impairment, use of hearing aids, presence of tinnitus, medication, noise exposure, pathologic changes in the ear, other relevant clinical manifestations, family history and consanguinity. The only inclusion criteria for our study were hearing loss and family history. Blood was drawn when subjects signed committee-approved consent forms for DNA extraction, and genomic DNA was extracted.

### Gene exclusion

All subjects were tested for *GJB2 *[4] by standard Sanger sequencing. The other eight deafness genes in the Jewish population have low prevalence and their known mutations were screened only in subjects manifesting a relevant phenotype or ethnic background. These genes include *GJB6 *[30], *PCDH15 *[31], *USH1C *[4], *MYO3A *[32], *SLC26A4 *[33], *POU4F3 *[34], the inverted duplication of *TJP2 *[35], and *LOXHD1 *[36]. All known deafness-causing mutations in the Palestinian population were excluded, including mutations in *CDH23, MYO7A, MYO15A, OTOF, PJVK, SLC26A4, TECTA, TMHS, TMPRSS3, OTOA, PTPRQ, and GPSM2 *[5-7].

### Capture libraries

Exons and the flanking 40 bp into introns of 246 human genes were selected for capture and sequencing. The 246 genes are listed in Additional file 1, and the target sequences are listed in Additional file 2. The exons were uploaded from both NIH (RefSeq) and UCSC databases, using the UCSC Genome Browser. These genes have been linked with hearing loss in humans or their orthologous genes have been associated with hearing loss in mice. We designed 3x tiling biotinylated cRNA 120-mer oligonucleotides to capture the selected sequences for Illumina paired-end sequencing, using the eArray algorithm, and these were purchased from Agilent Technologies (SureSelect Target Enrichment System).

Paired-end libraries were prepared by shearing 3 μg of germline DNA to a peak size of 200 bp using a Covaris S2. DNA was cleaned with AmpPure XP beads (which preferentially removes fragments < 150 bp), end repaired, A-tailed and ligated to Illumina indexing-specific paired-end adapters. The libraries were amplified for five cycles with flanking primers (forward primer PE 1.0 and reverse primer SureSelect Indexing Pre-Capture PCR). The purified amplified library (500 ng) was then hybridized to the custom biotinylated cRNA oligonucleotides for 24 hours at 65°C. The biotinylated cRNA-DNA hybrids were purified with streptavidin-conjugated magnetic beads, washed, and the cRNA probes were digested, following cleaning of the captured DNA fragments with AmpPure XP beads. Barcode sequences for multiplex sequencing were added to the captured DNA samples, and a post capture PCR was performed for 14 cycles. The libraries were prepared using reagents from Illumina (Genomic DNA Sample Preparation Kit and Multiplexing Sample Preparation Oligonucleotide Kit) and Agilent (SureSelect Target Enrichment System Kit), according to Agilent's instructions. The final concentration of each captured library was determined by a Qubit fluorometer and multiple aliquots diluted to 0.5 ng/μl were analyzed on a high sensitivity chip with a Bioanalyzer 2100.

### Massively parallel sequencing

A final DNA concentration of 12 pM was used to carry out cluster amplification on v4 Illumina flow cells with an Illumina cluster generator instrument. We used a 2 × 72-bp paired-end recipe plus a third read to sequence the 6-bp index to sequence 11 captured library samples in total (Table 1), multiplexed in 7 lanes (1 or 2 multiplexed samples per lane), on the Illumina Genome Analyzer IIx, following the manufacturer's protocol. After running the GERALD demultiplexing script (Illumina), approximately 8 Gb of passing filter reads were generated for samples loaded in pairs on the flow cell lanes, and approximately 16 and approximately 19 Gb were generated for samples CK3 and W1098 that were loaded alone, respectively. The reads were aligned to our BED file of bait probe (capture) targets, and reads that were not included in the captured sequences were discarded. The average on-target capture efficiency was 66%. The median base coverage was 757 × to 2,080 ×. Samples that were loaded alone on a lane had an average base coverage of 1970 ×, while samples loaded two in lane had an average base coverage of 937 ×. Overall, 94.7% of our targeted bases were covered by more than 10 reads, and 92% were covered by more than 30 reads, our cutoffs for variant detection. The remaining approximately 5% of the poorly covered regions (< 10 reads) were in extremely high GC-rich regions. Raw sequencing data are available at the EBI Sequence Read Archive (SRA) with accession number ERP000823.

### Bioinformatics

To identify SNPs and point mutations, data were aligned to hg19 using Burrows-Wheeler Aligner (BWA) [37] and MAQ [38], after removal of reads with duplicate start and end sites. BWA was also used to calculate average coverage per targeted base. SNP detection was performed using the SNP detection algorithms of MAQ and SNVmix2 [39]; the latter was also used to count the real number of variant and consensus reads for each SNP, to distinguish between heterozygote and homozygote variants. In addition, a read-depth algorithm was used to detect exonic deletions and duplications [40]. In order to sort potentially deleterious alleles from benign polymorphisms, perl scripts (available from the authors by request) were used to filter the variants (SNPs and indels) obtained against those of dbSNP132. Because dbSNP132 includes both disease-associated and benign alleles, known variants identified by NCBI were included only if clinically associated. The VariantClassifier algorithm [41] was used to add the following information for surviving variants: gene name, the predicted effect on gene (at or near splice site) and protein function (missense, nonsense, truncation), context (coding or non-coding sequence), and if it is in coding sequence, the amino acid change.

The Placental Mammal Basewise Conservation by PhyloP (phyloP46wayPlacental) score for the consensus nucleotide in each SNP was obtained from the UCSC Genome Browser, and variants with a score < 0.9 were considered as non-conserved and discarded from the SNP lists. Since we sequenced DNA samples of 11 probands from similar ethnic groups, we also counted the number of probands that carry each variant, finding many novel variants that are common in the Jewish and/or Palestinian ethnic groups, although not included in dbSNP132, which are most probably non-damaging variants. For variants of conserved nucleotides that present in up to three probands, we also checked if this variant was already reported in the 1000 Genomes project or in other published genomes from hearing humans.

The effect of rare or private non-synonymous SNPs was assessed by the PolyPhen-2 (Prediction of functional effects of human nsSNPs) HumVar score [42] and SIFT algorithm (Sorting Tolerant From Intolerant) [43], which predict damage to protein function or structure based on amino acid conservation and structural data. Although thousands of variants were detected in each proband (both SNPs and indels), this analysis yielded a small number of variants that may affect protein function.

### Sanger sequencing

Sequencing was performed using the ABI Prism BigDye Terminator Cycle Sequencing Ready Reaction Kit (Perkin-Elmer Applied Biosystems, Foster City, CA, USA) and an ABI 377 DNA sequencer.

### Restriction enzyme assays

For screening unrelated deaf individuals and population controls, restriction enzyme assays were designed for detection of *CDH23 *c.7903G > T (p.V2635F); *TMC1 *c.1810C > T (p.R604X), c.1939T > C (p.S647P) and c.1210T > C, W404R; *MYO15A *c.8183G > A (p.R2728H) and c.373delCG (p.R125VfsX101); and *TECTA *c.5597C > T (p.T1866M) (Additional file 4). PCR assays were used for *MYO15A *c.4240G > A (p.E1414K) and *WFS1 *c.2765G > A (p.E864K) (Additional file 4).

## Abbreviations

bp: base pair; indel: insertion-deletion; MPS: massively parallel sequencing; SNP: single nucleotide polymorphism.

## Authors' contributions

LMF, ZB, HS, MCK, TW, MK and KBA conceived and designed the experiments and analyses and wrote the paper. ZB, HS, DL, SS, MF, BD, MS, MR, SL, EL-L and MK ascertained the families, collected DNA samples, and assessed auditory function. LMF, ZB, HS, VOK, AAR, TP and TW performed laboratory experiments. LMF, NK, MKL, and NS carried out bioinformatics analyses. All authors read and approved the final manuscript.

## Supplementary Material

Additional file 1 **Table of human genes captured**. Click here for file

Additional file 2 **Table of captured sequences**. Click here for file

Additional file 3 **Tables of indels and SNPs in four or more probands in our population**. **(a) **Table of indels appearing in four or more probands in our population (*n *= 11). **(b) **Table of SNPs in four or more probands in our population (*n *= 11). Click here for file

Additional file 4 **Table of primers and restriction enzyme digestion assays**. Click here for file

## References

[B1] Van Camp G Smith RJH Hereditary Hearing Loss Homepage. 2011 http://hereditaryhearingloss.org

[B2] Nance WE The genetics of deafness. Ment Retard Dev Disabil Res Rev 2003 9 109 119 10.1002/mrdd.10067 12784229

[B3] Gorlin RJ Toriello HV Cohen MM Hereditary Hearing Loss and its Syndromes 1995 Oxford: Oxford University Press

[B4] Brownstein Z Avraham KB Deafness genes in Israel: implications for diagnostics in the clinic. Pediatr Res 2009 66 128 134 10.1203/PDR.0b013e3181aabd7f 19390476

[B5] Shahin H Walsh T Rayyan AA Lee MK Higgins J Dickel D Lewis K Thompson J Baker C Nord AS Stray S Gurwitz D Avraham KB King MC Kanaan M Five novel loci for inherited hearing loss mapped by SNP-based homozygosity profiles in Palestinian families. Eur J Hum Genet 2010 18 407 413 10.1038/ejhg.2009.190 19888295 PMC2987250

[B6] Walsh T Shahin H Elkan-Miller T Lee MK Thornton AM Roeb W Abu Rayyan A Loulus S Avraham KB King MC Kanaan M Whole exome sequencing and homozygosity mapping identify mutation in the cell polarity protein GPSM2 as the cause of nonsyndromic hearing loss *DFNB82*. Am J Hum Genet 2010 87 90 94 10.1016/j.ajhg.2010.05.010 20602914 PMC2896776

[B7] Shahin H Rahil M Abu Rayan A Avraham KB King MC Kanaan M Walsh T Nonsense mutation of the stereociliar membrane protein gene *PTPRQ *in human hearing loss *DFNB84*. J Med Genet 2010 47 643 645 10.1136/jmg.2009.075697 20472657

[B8] Gnirke A Melnikov A Maguire J Rogov P LeProust EM Brockman W Fennell T Giannoukos G Fisher S Russ C Gabriel S Jaffe DB Lander ES Nusbaum C Solution hybrid selection with ultra-long oligonucleotides for massively parallel targeted sequencing. Nat Biotechnol 2009 27 182 189 10.1038/nbt.1523 19182786 PMC2663421

[B9] Tewhey R Nakano M Wang X Pabon-Pena C Novak B Giuffre A Lin E Happe S Roberts DN LeProust EM Topol EJ Harismendy O Frazer KA Enrichment of sequencing targets from the human genome by solution hybridization. Genome Biol 2009 10 R116 10.1186/gb-2009-10-10-r116 19835619 PMC2784331

[B10] Verpy E Masmoudi S Zwaenepoel I Leibovici M Hutchin TP Del Castillo I Nouaille S Blanchard S Laine S Popot JL Moreno F Mueller RF Petit C Mutations in a new gene encoding a protein of the hair bundle cause non-syndromic deafness at the *DFNB16 *locus. Nat Genet 2001 29 345 349 10.1038/ng726 11687802

[B11] Sirmaci A Duman D Ozturkmen-Akay H Erbek S Incesulu A Ozturk-Hismi B Arici ZS Yuksel-Konuk EB Tasir-Yilmaz S Tokgoz-Yilmaz S Cengiz FB Aslan I Yildirim M Hasanefendioglu-Bayrak A Aycicek A Yilmaz I Fitoz S Altin F Ozdag H Tekin M Mutations in *TMC1 *contribute significantly to nonsyndromic autosomal recessive sensorineural hearing loss: a report of five novel mutations. Int J Pediatr Otorhinolaryngol 2009 73 699 705 10.1016/j.ijporl.2009.01.005 19187973

[B12] Kurima K Yang Y Sorber K Griffith AJ Characterization of the transmembrane channel-like (*TMC*) gene family: functional clues from hearing loss and epidermodysplasia verruciformis. Genomics 2003 82 300 308 10.1016/S0888-7543(03)00154-X 12906855

[B13] Labay V Weichert RM Makishima T Griffith AJ Topology of transmembrane channel-like gene 1 protein. Biochemistry 2010 49 8592 8598 10.1021/bi1004377 20672865 PMC3005947

[B14] Meyer CG Gasmelseed NM Mergani A Magzoub MM Muntau B Thye T Horstmann RD Novel *TMC1 *structural and splice variants associated with congenital nonsyndromic deafness in a Sudanese pedigree. Hum Mutat 2005 25 100 15605408 10.1002/humu.9302

[B15] Shearer AE Hildebrand MS Webster JA Kahrizi K Meyer NC Jalalvand K Arzhanginy S Kimberling WJ Stephan D Bahlo M Smith RJ Najmabadi H Mutations in the first MyTH4 domain of *MYO15A *are a common cause of *DFNB3 *hearing loss. Laryngoscope 2009 119 727 733 10.1002/lary.20116 19274735 PMC2885251

[B16] Liburd N Ghosh M Riazuddin S Naz S Khan S Ahmed Z Liang Y Menon PS Smith T Smith AC Chen KS Lupski JR Wilcox ER Potocki L Friedman TB Novel mutations of *MYO15A *associated with profound deafness in consanguineous families and moderately severe hearing loss in a patient with Smith-Magenis syndrome. Hum Genet 2001 109 535 541 10.1007/s004390100604 11735029

[B17] Eiberg H Hansen L Kjer B Hansen T Pedersen O Bille M Rosenberg T Tranebjaerg L Autosomal dominant optic atrophy associated with hearing impairment and impaired glucose regulation caused by a missense mutation in the *WFS1 *gene. J Med Genet 2006 43 435 440 16648378 10.1136/jmg.2005.034892 PMC2649014

[B18] Valero R Bannwarth S Roman S Paquis-Flucklinger V Vialettes B Autosomal dominant transmission of diabetes and congenital hearing impairment secondary to a missense mutation in the *WFS1 *gene. Diabet Med 2008 25 657 661 10.1111/j.1464-5491.2008.02448.x 18544103

[B19] Fukuoka H Kanda Y Ohta S Usami S Mutations in the *WFS1 *gene are a frequent cause of autosomal dominant nonsyndromic low-frequency hearing loss in Japanese. J Hum Genet 2007 52 510 515 10.1007/s10038-007-0144-3 17492394

[B20] Verhoeven K Van Laer L Kirschhofer K Legan PK Hughes DC Schatteman I Verstreken M Van Hauwe P Coucke P Chen A Smith RJ Somers T Offeciers FE Van de Heyning P Richardson GP Wachtler F Kimberling WJ Willems PJ Govaerts PJ Van Camp G Mutations in the human alpha-tectorin gene cause autosomal dominant non-syndromic hearing impairment. Nat Genet 1998 19 60 62 10.1038/ng0598-60 9590290

[B21] Sagong B Park R Kim YH Lee KY Baek JI Cho HJ Cho IJ Kim UK Lee SH Two novel missense mutations in the *TECTA *gene in Korean families with autosomal dominant nonsyndromic hearing loss. Ann Clin Lab Sci 2010 40 380 385 20947814

[B22] Hildebrand MS Morin M Meyer NC Mayo F Modamio-Hoybjor S Mencia A Olavarrieta L Morales-Angulo C Nishimura CJ Workman H Deluca AP Del Castillo I Taylor KR Tompkins B Goodman CW Schrauwen I Van Wesemael M Lachlan K Shearer AE Braun TA Huygen PL Kremer H Van Camp G Moreno F Casavant TL Smith RJ Moreno-Pelayo MA *DFNA8/12 *caused by *TECTA *mutations is the most identified subtype of non-syndromic autosomal dominant hearing loss. Hum Mutat 2011 32 825 834 10.1002/humu.21512 21520338 PMC3326665

[B23] Leibovici M Safieddine S Petit C Mouse models for human hereditary deafness. Curr Top Dev Biol 2008 84 385 429 19186249 10.1016/S0070-2153(08)00608-X

[B24] Dror AA Avraham KB Hearing loss: mechanisms revealed by genetics and cell biology. Annu Rev Genet 2009 43 411 437 10.1146/annurev-genet-102108-134135 19694516

[B25] Shearer AE DeLuca AP Hildebrand MS Taylor KR Gurrola J Scherer S Scheetz TE Smith RJ Comprehensive genetic testing for hereditary hearing loss using massively parallel sequencing. Proc Natl Acad Sci USA 2010 107 21104 21109 10.1073/pnas.1012989107 21078986 PMC3000272

[B26] Hildebrand MS Kahrizi K Bromhead CJ Shearer AE Webster JA Khodaei H Abtahi R Bazazzadegan N Babanejad M Nikzat N Kimberling WJ Stephan D Huygen PL Bahlo M Smith RJ Najmabadi H Mutations in *TMC1 *are a common cause of *DFNB7/11 *hearing loss in the Iranian population. Ann Otol Rhinol Laryngol 2010 119 830 835 21250555 10.1177/000348941011901207 PMC3058627

[B27] Hilgert N Smith RJ Van Camp G Forty-six genes causing nonsyndromic hearing impairment: which ones should be analyzed in DNA diagnostics? Mutat Res 2009 681 189 196 10.1016/j.mrrev.2008.08.002 18804553 PMC2847850

[B28] Walsh T Abu Rayan A Abu Sa'ed J Shahin H Shepshelovich J Lee MK Hirschberg K Tekin M Salhab W Avraham KB King MC Kanaan M Genomic analysis of a heterogeneous Mendelian phenotype: multiple novel alleles for inherited hearing loss in the Palestinian population. Hum Genomics 2006 2 203 211 16460646 10.1186/1479-7364-2-4-203 PMC3525152

[B29] Shahin H Walsh T Sobe T Abu Sa'ed J Abu Rayan A Lynch ED Lee MK Avraham KB King MC Kanaan M Mutations in a novel isoform of *TRIOBP *that encodes a filamentous-actin binding protein are responsible for *DFNB28 *recessive nonsyndromic hearing loss. Am J Hum Genet 2006 78 144 152 10.1086/499495 16385458 PMC1380212

[B30] Lerer I Sagi M Ben-Neriah Z Wang T Levi H Abeliovich D A deletion mutation in *GJB6 *cooperating with a *GJB2 *mutation in trans in non-syndromic deafness: A novel founder mutation in Ashkenazi Jews. Hum Mutat 2001 18 460 11668644 10.1002/humu.1222

[B31] Brownstein Z Ben-Yosef T Dagan O Frydman M Abeliovich D Sagi M Abraham FA Taitelbaum-Swead R Shohat M Hildesheimer M Friedman TB Avraham KB The R245X mutation of *PCDH15 *in Ashkenazi Jewish children diagnosed with nonsyndromic hearing loss foreshadows retinitis pigmentosa. Pediatr Res 2004 55 995 1000 10.1203/01.PDR.0000125258.58267.56 15028842

[B32] Walsh T Walsh V Vreugde S Hertzano R Shahin H Haika S Lee MK Kanaan M King MC Avraham KB From flies' eyes to our ears: mutations in a human class III myosin cause progressive nonsyndromic hearing loss *DFNB30*. Proc Natl Acad Sci USA 2002 99 7518 7523 10.1073/pnas.102091699 12032315 PMC124268

[B33] Brownstein ZN Dror AA Gilony D Migirov L Hirschberg K Avraham KB A novel *SLC26A4 (PDS*) deafness mutation retained in the endoplasmic reticulum. Arch Otolaryngol Head Neck Surg 2008 134 403 407 10.1001/archotol.134.4.403 18427006

[B34] Vahava O Morell R Lynch ED Weiss S Kagan ME Ahituv N Morrow JE Lee MK Skvorak AB Morton CC Blumenfeld A Frydman M Friedman TB King MC Avraham KB Mutation in transcription factor *POU4F3 *associated with inherited progressive hearing loss in humans. Science 1998 279 1950 1954 10.1126/science.279.5358.1950 9506947

[B35] Walsh T Pierce SB Lenz DR Brownstein Z Dagan-Rosenfeld O Shahin H Roeb W McCarthy S Nord AS Gordon CR Ben-Neriah Z Sebat J Kanaan M Lee MK Frydman M King MC Avraham KB Genomic duplication and overexpression of TJP2/ZO-2 leads to altered expression of apoptosis genes in progressive nonsyndromic hearing loss *DFNA51*. Am J Hum Genet 2010 87 101 109 10.1016/j.ajhg.2010.05.011 20602916 PMC2896780

[B36] Edvardson S Jalas C Shaag A Zenvirt S Landau C Lerer I Elpeleg O A deleterious mutation in the *LOXHD1 *gene causes autosomal recessive hearing loss in Ashkenazi Jews. Am J Med Genet A 2011 155A 1170 1172 21465660 10.1002/ajmg.a.33972

[B37] Li H Durbin R Fast and accurate short read alignment with Burrows-Wheeler transform. Bioinformatics 2009 25 1754 1760 10.1093/bioinformatics/btp324 19451168 PMC2705234

[B38] Li H Ruan J Durbin R Mapping short DNA sequencing reads and calling variants using mapping quality scores. Genome Res 2008 18 1851 1858 10.1101/gr.078212.108 18714091 PMC2577856

[B39] Goya R Sun MG Morin RD Leung G Ha G Wiegand KC Senz J Crisan A Marra MA Hirst M Huntsman D Murphy KP Aparicio S Shah SP SNVMix: predicting single nucleotide variants from next-generation sequencing of tumors. Bioinformatics 2010 26 730 736 10.1093/bioinformatics/btq040 20130035 PMC2832826

[B40] Walsh T Lee MK Casadei S Thornton AM Stray SM Pennil C Nord AS Mandell JB Swisher EM King MC Detection of inherited mutations for breast and ovarian cancer using genomic capture and massively parallel sequencing. Proc Natl Acad Sci USA 2010 107 12629 12633 10.1073/pnas.1007983107 20616022 PMC2906584

[B41] Li K Stockwell TB VariantClassifier: A hierarchical variant classifier for annotated genomes. BMC Res Notes 2010 3 191 10.1186/1756-0500-3-191 20626889 PMC2913924

[B42] Adzhubei IA Schmidt S Peshkin L Ramensky VE Gerasimova A Bork P Kondrashov AS Sunyaev SR A method and server for predicting damaging missense mutations. Nat Methods 2010 7 248 249 10.1038/nmeth0410-248 20354512 PMC2855889

[B43] Kumar P Henikoff S Ng PC Predicting the effects of coding non-synonymous variants on protein function using the SIFT algorithm. Nat Protoc 2009 4 1073 1081 10.1038/nprot.2009.86 19561590

